# Gene Expression Profiling Stratifies IDH-Wildtype Glioblastoma With Distinct Prognoses

**DOI:** 10.3389/fonc.2019.01433

**Published:** 2019-12-17

**Authors:** Yu-Qing Liu, Fan Wu, Jing-Jun Li, Yang-Fang Li, Xing Liu, Zheng Wang, Rui-Chao Chai

**Affiliations:** ^1^Department of Molecular Neuropathology, Beijing Neurosurgical Institute, Beijing, China; ^2^Chinese Glioma Genome Atlas Network, Beijing, China; ^3^Department of Neurosurgery, Beijing Tiantan Hospital, Capital Medical University, Beijing, China

**Keywords:** glioma, GBM, IDH wildtype, risk signature, biomarker, prognosis

## Abstract

**Objectives:** In the present study, we aimed to determine the candidate genes that may function as biomarkers to further distinguish patients with isocitrate dehydrogenase (IDH)-wildtype glioblastoma (GBM), which are heterogeneous with respect to clinical outcomes.

**Materials and Methods:** We selected 41 candidate genes associated with overall survival (OS) using univariate Cox regression from IDH-wildtype GBM patients based on RNA sequencing (RNAseq) expression data from the Chinese Glioma Genome Atlas (CGGA, *n* = 105) and The Cancer Genome Atlas (TCGA, *n* = 139) cohorts. Next, a seven-gene-based risk signature was formulated according to Least Absolute Shrinkage and Selection Operator (LASSO) regression algorithm in the CGGA RNAseq database as a training set, while another 525 IDH-wildtype GBM patient TCGA datasets, consisting of RNA sequencing and microarray data, were used for validation. Patient survival in the low- and high-risk groups was calculated using Kaplan-Meier survival curve analysis and the log-rank test. Uni-and multivariate Cox regression analysis was used to assess the prognosis value. Gene oncology (GO) and gene set enrichment analysis (GSEA) were performed for the functional analysis of the seven-gene-based risk signature.

**Results:** We developed a seven-gene-based signature, which allocated each patient to a risk group (low or high). Patients in the high-risk group had dramatically shorter overall survival than their low-risk counterparts in three independent cohorts. Univariate and multivariate analysis showed that the seven-gene signature remained an independent prognostic factor. Moreover, the seven-gene risk signature exhibited a striking prognostic validity, with AUC of 78.4 and 73.9%, which was higher than for traditional “age” (53.7%, 62.4%) and “GBM sub-type” (57.7%, 52.9%) in the CGGA- and TCGA-RNAseq databases, respectively. Subsequent bioinformatics analysis predicted that the seven-gene signature was involved in the inflammatory response, immune response, cell adhesion, and apoptotic process.

**Conclusions:** Our findings indicate that the seven-gene signature could be a potential prognostic biomarker. This study refined the current classification system of IDH-wildtype GBM and may provide a novel perspective for the research and individual therapy of IDH-wildtype GBM.

## Introduction

Glioblastoma (GBM, WHO grade IV) is the most common and malignant primary intracranial tumor and is associated with a poor prognosis, with a median survival rate of 14–16 months, despite the use of intensive treatments, including surgery, radiotherapy, and chemotherapy (1–4). Isocitrate dehydrogenase (IDH) mutations are one of the most common and earliest detectable genetic alterations in diffuse gliomas, and evidence supports this mutation as a driver of glioma genesis (5). Based on the updated 2016 edition of World Health Organization (WHO) classification of central nervous system (CNS) tumors, GBM could be classified into IDH-wildtype, and IDH-mutant GBM, wherein the former (IDH-wildtype GBM) is associated with a worse prognosis (6, 7). IDH-mutant GBM accounts for about 12% of all GBM, with an occurrence rate in secondary GBM of 84.6%, while its counterpart in primary GBM is rare (5.0%) (8, 9).

Previous studies have indicated that a six-gene signature could further stratify the prognosis of IDH-mutant glioma using gene expression profiling (10). Given that IDH-wildtype and IDH-mutant GBM are regarded as distinct entities despite their similar histology (11), further stratification of patients with IDH-mutant or IDH-wildtype GBM could be a promising approach for the diagnosis and treatment of GBM. The methylation status of the methylguanine methyltransferase (MGMT) promoter has been reported to have predictive value for both IDH-mutant and IDH-wildtype GBM (12, 13). Our recent study presented a comprehensive somatic mutation landscape of secondary GBM and provided a protocol for MET-targeted therapy for precision neuro-oncology (14). A handful of recent studies have assessed the molecular spectrum of IDH-mutant GBM on the basis of genome-wide DNA methylation analysis, copy-number profiling, and gene expression profiling, respectively (11, 15). Nevertheless, the systematical investigation of IDH-wildtype GBM (88%), which is overwhelmingly more common than for IDH-mutant GBM, as well as being heterogeneous with respect to clinical outcomes, remains to be discussed completely.

In the present study, we aimed to further analyze and stratify IDH-wildtype GBM assessed by whole-genome expression profile analysis. We identified a seven-gene-based risk signature for IDH-wildtype GBM in the CGGA-RNAseq cohort, which was then validated in TCGA-RNAseq and TCGA-microarray cohorts. Furthermore, the prognostic value of our signature and underlying biological functions correlated with this signature were also systemically investigated. By improving our understanding of the molecular basis of IDH-wildtype GBM, we expect to develop a superior stratification of these tumors according to the risk signature and supply additional therapeutic targets for the treatment of for IDH-wildtype GBM.

## Materials and Methods

### Samples and Data Collection

This study collected 630 GBM samples from three cohorts: the CGGA-RNA sequencing (RNAseq, *n* = 105), TCGA-RNAseq (*n* = 139), and TCGA-microarray (*n* = 386) cohorts. The clinical and molecular information of the cases in the CGGA-RNAseq cohort were obtained from the CGGA database@@uline (http://www.cgga.org.cn/) and were used as the training set (16). Each case with newly diagnosed GBM was treated by the CGGA group. All tissues were diagnosed histologically by two or more neuropathologists, independently. The overall survival (OS) was calculated from the date of diagnosis until the death of the patient or the end of the clinical follow-up. The study protocol was approved by the Ethics Committee of the Beijing Tiantan Hospital. Another 525 GBM were included from TCGA-RNAseq and TCGA-microarray cohorts (https://tcga-data.nci.nih.gov/tcga/) (17, 18) as validation sets. The characteristics of the patient in the three cohorts are provided in Table 1.

**Table 1:** Clinicopathological characteristics of patients in the CGGA RNAseq, TCGA RNAseq, and TCGA microarray cohorts.

**Characteristics**		**CGGA RNAseq cohort**	**TCGA RNAseq cohort**	**TCGA microarray cohort**
		**GBM-IDH wildtype (*n* = 105)**	**GBM-IDH wildtype (*n* = 139)**	**GBM-IDH wildtype (*n* = 386)**
Age (years)	Median (range)	52 (8–81)	62 (24–89)	61 (19–89)
	Age≥45	35 (33.3%)	11 (7.9%)	43 (11.1%)
	Age <45	70 (66.7%)	128 (92.1%)	343 (88.9%)
Gender	Male	68 (64.8%)	89 (64.0%)	239 (61.9%)
	Female	37 (35.2%)	50 (36.0%)	147 (38.1%)
GBM sub-type	Proneural	5 (4.8%)	24 (17.3%)	72 (18.7%)
	Neural	10 (9.5%)	5 (3.6%)	70 (18.1%)
	Classical	41 (39.0%)	85 (61.1%)	108 (28.0%)
	Mesenchymal	49 (46.7%)	25 (18.0%)	115 (29.8%)
	NA	0 (0.00%)	0 (0.00%)	21 (5.4%)
MGMT promoter methylation status	Methylated	37 (35.2%)	43 (30.9%)	122 (31.6%)
	Unmethylated	65 (61.9%)	67 (48.2%)	151 (39.1%)
	NA	3 (2.9%)	29 (20.9%)	113 (29.3%)
TERT promoter status	Wildtype	49 (46.7%)	NA	NA
	Mutation	33 (31.4%)	NA	NA
	NA	23 (21.9%)	NA	NA
EGFR	Wildtype	NA	104 (74.8%)	NA
	Mutation	NA	34 (24.5%)	NA
	NA	NA	1 (0.7%)	NA
TP53	Wildtype	NA	106 (76.3%)	NA
	Mutation	NA	32 (23.0%)	NA
	NA	NA	1 (0.7%)	NA
Chr 7 gain/Chr 10 loss	Combined	NA	93 (66.9%)	NA
	No combined	NA	42 (30.2%)	NA
	NA	NA	4 (2.9%)	NA

*CGGA, Chinese Glioma Genome Atlas; TCGA, The Cancer Genome Atlas; MGMT, methylguanine methyltransferase; TERT, telomerase reverse transcriptase; NA, not applicable; Chr, chromosome*.

### Gene Oncology (GO), Kyoto Encyclopedia of Genes and Genomes (KEGG), and Gene Set Enrichment Analysis (GSEA)

GO and KEGG pathway analyses were performed in DAVID (http://david.abcc.ncifcrf.gov/home.jsp) for the functional annotation of the genes correlated positively and negatively with the risk score in the two cohorts (19). GO was used to analyze the main function of the differential expression genes. KEGG was performed to analyze pathway enrichment. GSEA was performed to determine whether the gene sets were statistically different between the two groups (high-risk score vs. low-risk score) using GSEA v3 software (http://www.broadinstitute.org/gsea/index.jsp) (20).

### Statistical Analysis

Univariate Cox regression analysis was performed to select genes associated with the OS in the CGGA-RNAseq and TCGA-RNAseq cohorts, respectively. Next, the risk associated genes (HR >1) and protective genes (HR <1) were both overlapped between the two cohorts. Ultimately, a total of 41 OS-correlated genes, consisting of 34 risk associated genes and 7 protective genes, were selected to perform further gene signature selection and risk-based classification in the training dataset. A risk signature was formulated according to Least Absolute Shrinkage and Selection Operator (LASSO) regression algorithm (21–25). The penalty parameter λ was chosen based on a 50-fold cross validation within the training dataset, which produced the minimum mean cross-validated error for the Cox model. Accordingly, seven genes and their regression coefficients were achieved. The risk score was then calculated in the training and validation datasets using the following equation:

$$\begin{array}{r} Risk\;score=\overset{n}{\underset{i=1}{\sum }}Coef_{i}\;\times \;Expr_{i}\; \end{array}$$

where *Coef*_*i*_ is the coefficient and *Expr*_*i*_ is the z-score-transformed relative expression value of each selected gene. Based on the median risk value (23, 26), patients in CGGA-RNAseq, TCGA-RNAseq, and TCGA microarray databases were divided into high- and low-risk groups. Kaplan-Meier survival curves were calculated and the log rank tests were conducted to assess the prognostic significance (13, 27). Differences in clinicopathologic features between the groups were determined using the Student's t- or Chi-square tests. Multivariate Cox regression analyses were used to confirm independent prognostic factors. All statistical analyses were performed using SPSS version 16.0 software (SPSS Inc., Chicago, IL). *P*-value < 0.05 was considered as statistically significant.

## Results

### Screening for Critical Genes Stratified for IDH-Wildtype GBM Through Gene Expression

**Figure 1:**
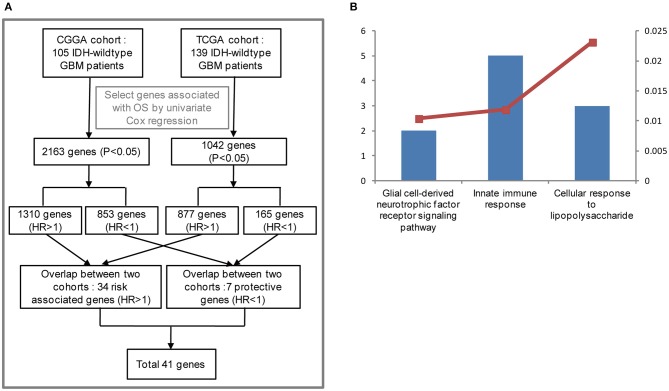
Functional analysis of 41 candidate genes associated with overall survival (OS). **(A)** Flowchart of data analysis for the search of OS-correlated critically important genes. The GBM IDH-wild type patients from the CGGA (105) and TCGA cohorts (139) were analyzed. As a result, 2,163 and 1,042 genes associated with OS were selected by univariate Cox regression, respectively. Among the 2,163 genes for the CGGA cohort, 1,310 genes were considered to be risk associated genes (HR >1) and 853 genes were identified to be protective genes (HR<1). As for the 1,042 genes from the TCGA cohort, 877 genes (HR >1), and 165 genes (HR<1) were regarded as risk associated and protective genes, respectively. The risk associated and protective genes were then overlapped between the CGGA and TCGA cohorts, respectively. As a result, 41 candidate genes associated with OS were obtained by combining 34 risk associated genes with 7 protective genes. **(B)** Functional annotation of 41 candidate genes using GO terms of biological processes. The left bar represents the gene number and the right bar represents the *P*-value. CGGA, Chinese Glioma Genome Atlas; TCGA, The Cancer Genome Atlas; HR, hazard ratio; OS, overall survival.

To stratify IDH-wildtype GBM based on the whole genome expression profiling, we firstly selected genes associated with overall survival (OS) using univariate Cox regression from IDH-wildtype GBM patients in the CGGA (*n* = 105) and TCGA cohorts (*n* = 139), respectively. Next, the OS-related genes in the two cohorts were divided into two groups: protective genes (HR <1) and risk associated genes (HR >1). The protective genes and risk associated genes were then overlapped between the two cohorts, respectively. Finally, 41 candidate genes, including 34 risk associated genes and 7 protective genes, were selected (Figure 1A). The functional annotations of the 41 candidate genes were enriched in GO terms for biological processes including “Innate immune response,” “Glial cell-derived neurotrophic factor receptor signaling pathway,” and “Cellular response to lipopolysaccharide” (Figure 1B).

### Identification of a Seven-Gene Risk Signature for IDH-Wildtype GBM

**Figure 2:**
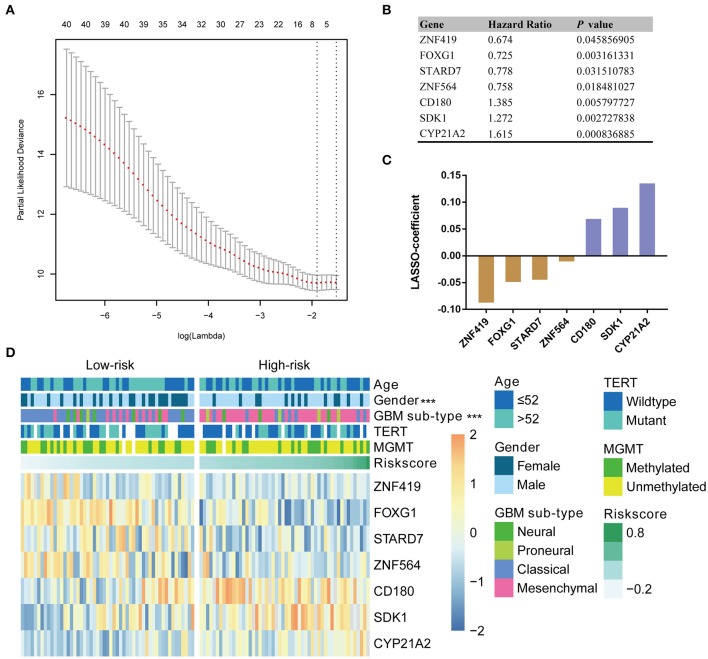
Identification of a seven-gene risk signature for OS by LASSO regression analysis in CGGA GBM-IDH wildtype datasets. **(A)** Partial likelihood deviance as a function of the regularization parameter λ in the CGGA GBM-IDH wildtype dataset. The red point denotes the λ value along the regularization paths, and the gray error bars represent the confidence intervals for the cross-validated error rate. The horizontal row of numbers above the plot denotes the gene number in each condition upon shrinkage and selection based on linear regression. The left vertical dotted line denotes the minimum error, and the right vertical dotted line represents the largest λ value. The gene expression analyses of seven genes selected and their regression coefficients by LASSO are shown in **(B,C)**, respectively. **(D)** Heat map showing the association between the risk scores and clinicopathological features based on the seven-gene risk signature. CGGA, Chinese Glioma Genome Atlas; LASSO, Least Absolute Shrinkage and Selection Operator; MGMT, methylguanine methyltransferase; TERT, telomerase reverse transcriptase; TCGA, The Cancer Genome Atlas; HR, hazard ratio.

Given the prognostic importance of these 41 candidate genes, we attempted to develop a gene expression-based signature that could further stratify IDH-wild type GBM derived from these genes. To this end, using LASSO regression algorithm, seven genes, including 4 protective (*ZNF419, FOXG1, STARD7*, and *ZBTB16*) genes and 3 risk associated genes (*CD180, SDK1*, and *CYP21A2*), were selected as active covariates to assess their prognostic value, thereby obtaining the risk scores for the patients in the training cohort (Figures 2A–C).

**Figure 3:**
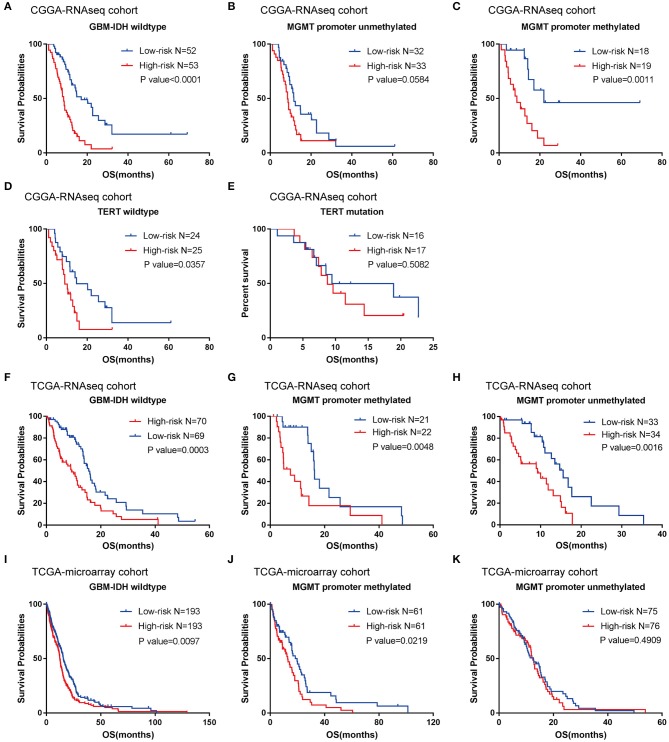
Prognostic significance of the seven-gene signature-based risk scores in GBM-IDH wildtype samples from CGGA and TCGA-RNAseq datasets. **(A–E)** Prognosis efficiency of the seven-gene risk signature in the total GBM-IDH wildtype **(A)**, and MGMT promoter unmethylated **(B)** and methylated **(C)**, TERT promoter wildtype **(D)**, and mutation samples **(E)** from the CGGA-RNAseq datasets, respectively. **(F–H)** Prognosis efficiency of the seven-gene risk signature in TCGA GBM-IDH wildtype datasets **(F)** and MGMT promoter unmethylated **(G)** and methylated **(H)** of GBM-IDH wildtype from the TCGA-RNAseq datasets, respectively. **(I–K)** Prognosis efficiency of the seven-gene risk signature in IDH wildtype GBM from TCGA-microarray datasets **(I)** and MGMT promoter unmethylated **(J)** and methylated **(K)** from the CGGA GBM-IDH wildtype datasets, respectively. The *P*-value shown in each panel were determined using a log-rank test between the two groups. *P* < 0.05 was considered as statistically significant. CGGA, Chinese Glioma Genome Atlas; GBM, glioblastoma; OS, overall survival; TCGA, The Cancer Genome Atlas; IDH, isocitrate dehydrogenases.

According to their median risk score, patients were assigned to either a low- or high-risk group. In GBM with IDH-wildtype, Kaplan-Meier analysis showed that patients in the high-risk group (*n* = 53) had a lower OS than patients in the low-risk group (*n* = 52) in the training cohort (median OS = 8.47 vs. 17.13 months; *P* < 0.0001; Figure 3A). Furthermore, we validated the prognostic value of the risk score in the TCGA-RNAseq cohort. Consequently, we found that OS differed significantly between the high-risk (*n* = 70) and low-risk groups (*n* = 69) in IDH-wildtype GBM patients in the TCGA cohort (median OS = 9.27 vs. 15.57 months; *P* = 0.0003; Figure 3F).

Moreover, given that glioma sub-types are stratified according to the MGMT promoter methylation status, wherein telomerase reverse transcriptase (TERT) status showed distinct tumor characteristics and OS outcomes, we investigated the prognostic value of risk score in these populations. A similar trend was observed in these patients, although no significant difference was found in the unmethylated MGMT promoter or TERT mutation patients (most likely due to the small sample size) (Figures 3B–E,G,H). In summary, our results indicated that the high-risk group was markedly correlated with an unfavorable prognosis in patients with IDH-wildtype GBM.

To further validate the prognostic value of the seven-gene-based risk signature in other cohorts, we computed the risk scores for each patient in the TCGA microarray databases with the same formula. Patients were divided into low- and high-risk groups according to their median risk value. The survival analysis suggested that patients in the high-risk group (*n* = 193) had a lower OS than patients in the low-risk group (*n* = 193; median OS = 12.4 vs. 15.57 months; *P* = 0.0097; Figure 3I). For the group with a methylated MGMT promoter, compared to the low-risk patients, the OS of high-risk patients was also significantly lower (*P* = 0.0219; Figure 3J). On the other hand, there were no significant differences between patients with a methylated MGMT promoter (*P* >0.05; Figure 3K).

Subsequently, we explored whether the prognostic value of the seven-gene signature could be extended to IDH-mutant GBM and lower grade glioma (LGG, WHO grade II-III), by calculating a risk score using the same formula in the CGGA and TCGA cohorts. A Kaplan-Meier analysis showed that there was no significant difference between the low- and high-risk groups in IDH-mutant GBM from the CGGA-RNAseq and TCGA-microarray datasets (Figures S1A,B). According to the WHO 2016 update to the classification strategy, LGG were categorized into three subtypes (IDH-wildtype LGG, LGG-IDHmut- non-codel and LGG-IDHmut-codel) based on the status of IDH mutation and 1p/19q codeletion (7). The results showed that the survival time of the high-risk group was remarkably shorter than that of the low-risk group in LGG-IDHmut-non-codel (Figure S1D), whereas there was no significant difference in IDH-wildtype LGG and LGG-IDHmut-codel between the two risk groups in the CGGA-RNAseq cohort (Figures S1C,E). Furthermore, we found there was no significant difference in the survival of the high- and low-risk groups in all three subtypes of LGG from the TCGA-RNAseq cohort (Figures S1F–H). In summary, these results indicated that the seven-gene signature could not predict the prognosis of patients with IDH-mutant GBM and LGG, identified as an exclusive prognostic marker for IDH-wildtype GBM.

### Seven-Gene Signature Is an Independent Prognostic Factor for IDH-Wildtype GBM

**Figure 4:**
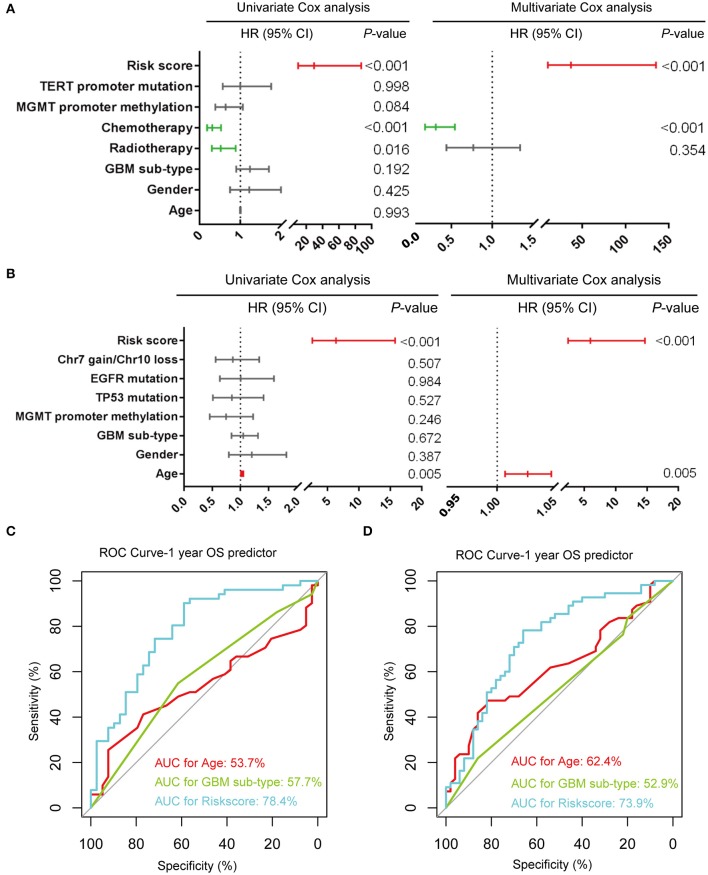
Prognostic validity of the seven-gene signature-based risk scores in GBM-IDH wildtype samples from CGGA and TCGA-RNAseq datasets. **(A,B)** Uni-and multivariate Cox regression analysis of the clinical features and seven-gene-based risk score for OS in GBM-IDH wildtype from CGGA **(A)** and TCGA **(B)** datasets. Variables with prognostic significance in univariate Cox regression analysis were included in further multivariate Cox analysis. Gender (female and male); GBM sub-type (neural, proneural, mesenchymal, and classical); MGMT promoter methylation status (methylated and unmethylated); TERT promoter status (mutant and wildtype); TP53 status (mutant and wildtype); EGFR status (mutant and wildtype); Chr7 gain/Chr10 loss status (combined and not combined); radiotherapy (yes and no); chemotherapy (yes and no); risk score (low and high). **(C,D)** Comparison between the seven-gene signature and traditional risk factors such as age and GBM sub-type in terms of sensitivity and specificity for predicting 1-year survival in the CGGA **(C)** and TCGA **(D)** datasets, respectively. CI, confidence interval; CGGA, Chinese Glioma Genome Atlas; HR, hazard ratio; OS, overall survival; ROC, receiver operating characteristic.

We further evaluated the prognostic value of the seven-gene signature for IDH-wildtype GBM patients. Uni- and multivariate Cox regression analyses of the clinical features and seven-gene-based risk score for OS were performed to determine the prognostic significance of the seven-gene signature in IDH-wildtype GBM patients from the CGGA datasets. The results showed that the seven-gene signature was independently associated with OS by adjusting for clinicopathological factors (age, gender, GBM sub-type, radiotherapy, chemotherapy, MGMT promoter methylation status, and TERT status; *P* < 0.001; Figure 4A). Consistently, the seven-gene risk signature was validated as an independent indicator after multivariate Cox regression analyses in the TCGA cohort (*P* < 0.001; Figure 4B).

### Prognostic Validity of the Seven-Gene Signature for IDH-Wildtype GBM

Subsequently, we determined the specificity and sensitivity of the risk score in the prediction of 1-year survival by calculating the area under the curve (AUC) of the risk score and the pathologic features using the receiving operator characteristic (ROC) curve. As shown in Figures 4C,D, the seven-gene signature showed a striking prognostic validity, with an AUC of 78.4 and 73.9%, which were higher those found for the traditional “age” (53.7%, 62.4%) and “GBM sub-type” (57.7%, 52.9%) in the CGGA- and TCGA-RNAseq databases, respectively. These data indicate that the seven-gene signature could be used as a potential prognostic marker of IDH-wildtype GBM.

### Association of the Seven-Gene Signature With Other Clinicopathological Features of IDH-Wildtype GBM

To evaluate the performance of the identified signature as a classifier, we classified the CGGA dataset into low- and high-risk groups using the median risk score as a cutoff point, and found a significant difference in several clinical characteristics between the two groups (Figure 2D). We found that male patients accounted for a large proportion, 75.5% of the total, of the high-risk group, compared to a proportion of male patients of 53.8% in the low-risk group (*P* < 0.001). As shown in Table S1, the classical and mesenchymal subtypes were found in 61.5 and 23.1%, 17.0 and 69.8% of low-risk and high-risk groups, respectively (*P* < 0.001) (Table S1).

In the TCGA-RNAseq cohort, patients were divided into low- and high-risk groups based on their median risk value. A marked difference was found in several molecular features between the two groups (Figure S2). The combination of “gain of chromosome 7” and “loss of chromosome 10” was found in 78.3 and 55.7% of patients in the low-risk and high-risk groups, respectively (*P* < 0.001). Moreover, we found that 30.4 and 18.6% of samples in the low- and high-risk groups, respectively, were found to harbor mutations in the epidermal growth factor receptor (EGFR) gene (*P* = 0.029) (Table S2). In conclusion, for the CGGA and TCGA cohorts, in comparison with the low-risk group, the high-risk group tended to consist of patients with a worse prognosis.

A previous study identified four clinically relevant subtypes (neural, proneural, classical, mesenchymal) of GBM using integrated genomic analysis (28). Here, we investigated the association between the seven-gene signature and subtype, and found that patients with mesenchymal GBM had a higher risk score than those with classical GBM in the CGGA (*P* < 0.0001; Figure S3A) and TCGA cohorts (*P* < 0.05; Figure S3E). On the other hand, there was no significant correlation between the risk signature and other features, such as age, MGMT status, TERT status, and P53 status in the CGGA (Figures S3B–D) and TCGA cohorts (Figures S3F–H).

### Functional Annotation of the Seven-Gene Signature

**Figure 5:**
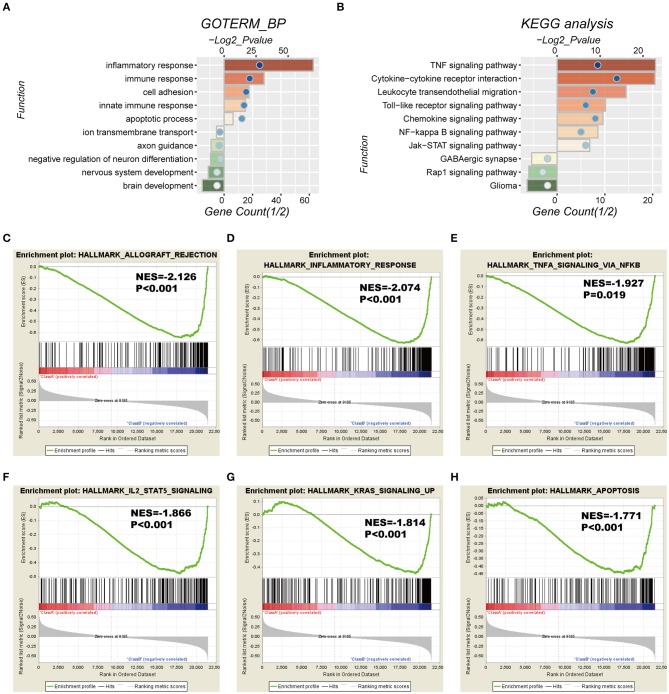
Functional characteristics correlated with the seven-gene signature in CGGA-RNAseq datasets. **(A,B)** Functional annotation of genes positively (red bar chart) or negatively (green bar chart) correlated with the risk score using the GO terms of biological processes **(A)** and the KEGG pathway **(B)**. Orange and green bars represent the *P*-value, and the blue dots represent the 1/2 gene count. **(C–H)** Gene set enrichment analysis (GSEA) shows that a higher risk score was positively associated with the inflammatory response, immune response-related signaling pathways, and apoptosis. NES, normalized enrichment score.

To investigate the potentially altered functional features correlated with the seven-gene signature, GO and KEGG analyses were conducted based on 589 high-risk score positively-related genes (*P* < 0.05) and 152 negatively-related genes (*P* < 0.05) using Pearson correlation analysis. GO enrichment showed that the top five involved biological processes, that is upregulated gene- in the high-risk group, were “inflammatory response,” “immune response,” “cell adhesion,” “innate immune response,” and “apoptotic process.” In contrast, downregulated genes in the high-risk group were closely associated with neurogenesis functions, such as “brain development,” “nervous system development,” “axon guidance,” and “ion transmembrane transport” (Figure 5A).

Moreover, KEGG pathway analysis showed that positively-related genes in the high-risk group were primarily enriched in biological processes for “TNF signaling pathway,” “cytokine-cytokine receptor interaction,” “leukocyte transendothelial migration,” “toll-like receptor signaling pathway,” and “chemokine signaling pathway,” whereas the negatively correlated genes were enriched in biological terms including “GABAergic synapse,” “Rap1 signaling pathway,” and “glioma” (Figure 5B).

In addition, GSEA analyses were performed for validation, showing that the high-risk groups were positively associated with inflammatory response (*P* < 0.001) and TNFα signaling via NFκB (*P* = 0.019), IL2-STAT5 signaling (*P* < 0.001), K-ras signaling (*P* < 0.001), and apoptosis (*P* < 0.001; Figures 5C–H). Consistently, these results were validated in the TCGA cohort (Figure S4).

## Discussion

In the current study, we studied various candidate genes with potential functions as biomarkers for the stratification of IDH-wildtype GBM with distinct prognoses using whole-genome expression data. We first screened 41 candidate genes, closely associated with OS of IDH-wildtype GBM, by combining the CGGA-RNAseq and TCGA-RNAseq datasets. We then created a seven-gene-based risk signature for IDH-wildtype GBM in the CGGA-RNAseq cohort, which was subsequently validated in the TCGA-RNAseq and TCGA-microarray cohorts. Moreover, the seven-gene risk signature, identified as an independent prognostic significance for IDH1-wildtype GBM, exhibited a greater prognostic value than other factors, underscoring the superiority of a gene expression profile-based signature (29, 30). Finally, bioinformatics analysis was used to predict that the seven-gene signature was involved in the inflammatory response, immune response, cell adhesion, and apoptotic process. To summarize, our seven-gene-based signature refined the current classification system of IDH-wildtype GBM and contributed to improving our understanding of the carcinogenesis and development of IDH-wildtype GBM.

In this study, we established a seven-gene-based signature based on the diversity of genes, including protective (*ZNF419, FOXG1, STARD7*, and ZBTB16) and risk associated (*CD180, SDK1*, and *CYP21A2*) genes, which could classify IDH-wildtype GBM into low- and high-risk groups to distinguish between the clinical outcomes. Among these genes, several had been previously studied in various tumors. Some studies have suggested that FoxG1 functions as an oncogene by promoting proliferation, as well as inhibiting differential responses in glioblastoma, by downregulating FoxO/Smad signaling (31). Moreover, low FoxG1 and high Olig-2 labeling indices define a prognostically favorable subset in IDH-mutant gliomas (32). ZBTB16 (Zinc Finger and BTB Domain Containing 16), is a transcription factor involved in the regulation of diverse biological processes, including cell proliferation, differentiation, organ development, stem cell maintenance, and innate immune cell development. A number of recent studies have now implicated PLZF in cancer progression as a tumor suppressor (33). CYP21A2 (21-hydroxylase) is a steroidogenic enzyme crucial for the synthesis of mineralocorticoids and glucocorticoids, and was identified as the most frequently mutated gene in esophageal squamous cell carcinoma by whole exome sequencing (34, 35).

The 2016 WHO classification made a clear difference between GBM that were IDH-mutant and those that were IDH-wildtype, and IDH-wildtype GBM carried a worse prognosis. Consistently, in the cohorts used in this study, the median OS of the IDH-wildtype GBM are 11.5, 13.3, and 13.83 months in the CGGA-RNAseq cohort, TCGA-RNAseq cohort and TCGA-microarray cohort, respectively, while, those of the IDH-mutant GBM are 18.5 months (CGGA-RNAseq cohort), 34.13 months (TCGA-RNAseq cohort), and 35.9 months (TCGA microarray cohort). However, we observed that the survival of patients with IDH-wildtype GBM varies from <3 months to more than 3 years, and the similar findings have also been reported in the previous study (3). Therefore, to further stratify IDH-wildtype GBM becomes important and meaningful.

In this study, we determined a seven-gene-based risk signature, a useful tool for risk stratification, to distinguish between prognoses for IDH-wildtype GBM in three independent cohorts. The median OS of patients with low- and high-risk are significantly different in the CGGA-RNAseq (17.13 vs. 8.47 months), TCGA-RNAseq (15.57 vs. 9.27 months) and TCGA-microarray cohorts (15.57 vs. 12.4 months). This is very meaningful to the post-operative management of these patients with IDH-wildtype GBM. Our previous study presented a gene signature based on GBM stem-like cell relevant genes for primary GBM (36). In addition, several previous studies have found a local immune signature for GBM, indicating the relationship between prognosis and the local immune response (19). Meanwhile, the strength of our study was based on the systematical expression profiling, the robust nature of risk score method (37), and validation across multi-platforms and multi-populations. Although the predictive value of the seven-gene signature was confirmed in distinct datasets, a prospective study with a larger sample size will be needed to assess its clinical relevance.

In conclusion, our results indicate that the seven-gene signature could be a potential prognostic biomarker, providing a novel perspective for research and treatment of IDH-wildtype GBM.

## Data Availability Statement

Publicly available datasets were analyzed in this study. This data can be found here: http://www.cgga.org.cn/.

## Ethics Statement

The study protocol was approved by the Ethics Committees of participating hospitals. All patients provided written and informed consent.

## Author Contributions

Y-QL and R-CC designed the study. Y-QL and FW reviewed the literature, created the figures and tables, and were responsible for the writing and critical editing of the manuscript. J-JL, Y-FL, XL, and ZW contributed to the data collection and analysis, as well as the critical revision of the manuscript. R-CC supervised the critical revision of the manuscript.

### Conflict of Interest

The authors declare that the research was conducted in the absence of any commercial or financial relationships that could be construed as a potential conflict of interest.
