# Supplementary material for: Gene Expression Profiling Stratifies IDH-Wildtype Glioblastoma With Distinct Prognoses
Source: Front Oncol. 2019 Dec 17;9:1433. doi: 10.3389/fonc.2019.01433 (PMC6929203; doi:10.3389/fonc.2019.01433)
Supplement: Supplementary file 1 [file Data_Sheet_1.docx]

**Figure S1. Prognostic value of the seven-gene signature in IDH-mutant GBM and lower grade gliomas (LGG, WHO grade II-III) in the CGGA and TCGA cohorts.**

**
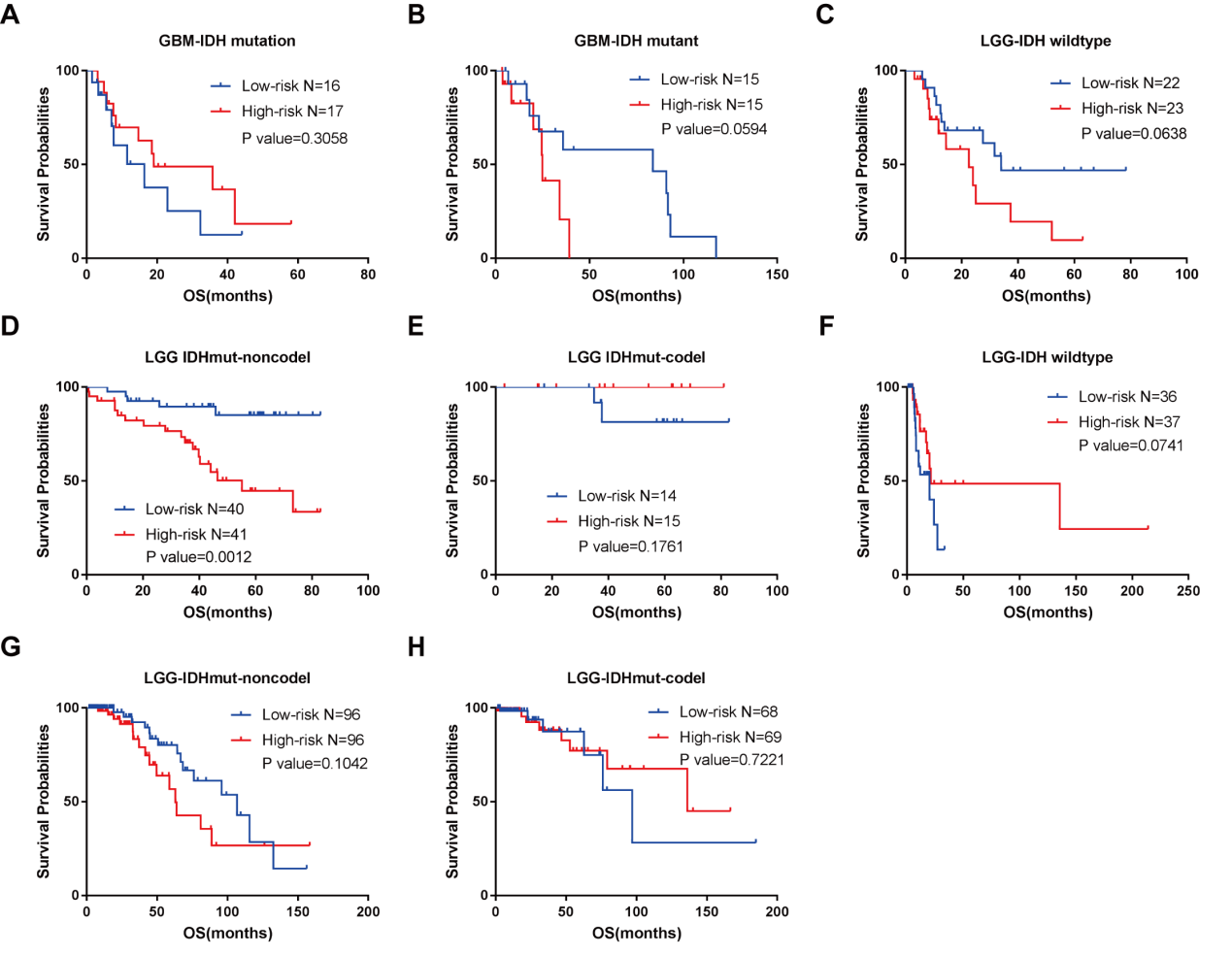
**

**Figure S1. Prognostic value of the seven-gene signature in IDH-mutant GBM and lower grade gliomas (LGG, WHO grade II-III) in the CGGA and TCGA cohorts. A, B.** Prognostic value of the seven-gene risk signature in IDH-mutation GBM from the CCGA-RNAseq **(A)** and TCGA-microarray datasets **(B)**, respectively. **C-H.** Prognosis efficiency of the seven-gene risk signature in IDH-wildtype LGG **(C, F),** LGG-IDH mutant and 1p/19q non-codeleted **(D, G)**, and LGG-IDH mutant and 1p/19q codeleted **(E, H)** from the CGGA- and TCGA-RNAseq datasets, respectively. *P*-value shown in each panel is determined by a log-rank test between the two groups. CGGA, Chinese Glioma Genome Atlas; GBM, glioblastoma; LGG, lower grade glioma; OS, overall survival; TCGA, The Cancer Genome Atlas; IDH, Isocitrate dehydrogenases.

**Table S1. Correlation between seven-gene based risk score and clinicopathologic factors of glioma patients in CGGA cohort.**

|  |  |  |  |
| --- | --- | --- | --- |
|  | Training set CGGA RNA-seq cohort (n = 105) | | |
| Features | Low-risk score (n=52) | High-risk score (n=53) | P-value |
| Age |  |  | 0.157 |
| Mean (range) | 54 (8-81) | 49 (12-71) |  |
| Gender |  |  | <0.001 |
| Female | 24(46.2%) | 13(24.5%) |  |
| Male | 28(53.8%) | 40(75.5%) |  |
| GBM sub-type |  |  | <0.001 |
| Proneural | 1(1.9%) | 4(7.5%) |  |
| Neural | 7(13.5%) | 3(5.7%) |  |
| Classical | 32(61.5%) | 9(17.0%) |  |
| Mesenchymal | 12(23.1%) | 37(69.8%) |  |
| TERT promoter status |  |  | 0.163 |
| Widetype | 21(40.4%) | 28(52.8%) |  |
| Mutation | 19(36.5%) | 14(26.4%) |  |
| NA | 12(23.1%) | 11(20.8%) |  |
| MGMT promoter methylation status |  |  | 0.517 |
| Unmethylated | 31(59.6%) | 34(64.2%) |  |
| Methylated | 19(36.5%) | 18(34.0%) |  |
| NA | 2(3.8%) | 1(1.9%) |  |

CGGA, Chinese Glioma Genome Atlas; TCGA, The Cancer Genome Atlas; MGMT, methylguanine methyltransferase; TERT, telomerase reverse transcriptase; NA, not applicable.

**Figure S2. Heat map shows the association of risk scores and clinicopathologic features based on the seven-gene risk signature in the TCGA dataset.**

**
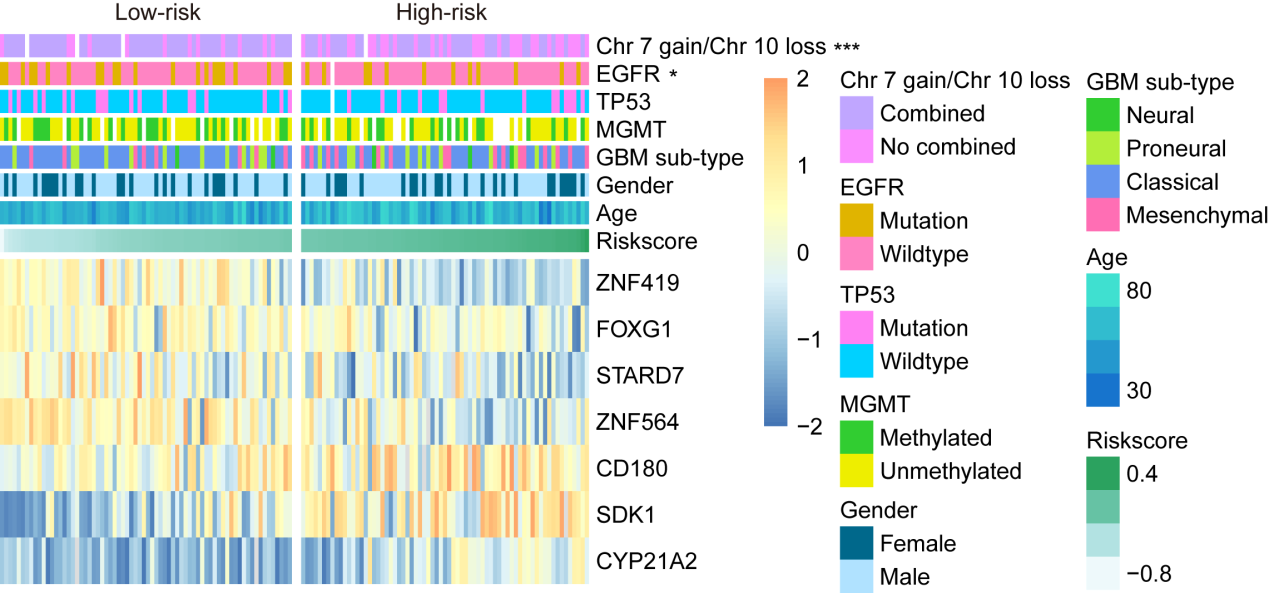
**

**Figure S2. Heat map shows the association of risk scores and clinicopathologic features based on the seven-gene risk signature in the TCGA dataset.**

**Table S2. Correlation between seven-gene based risk score and clinicopathologic factors of glioma patients in TCGA cohort.**

|  |  |  |  |
| --- | --- | --- | --- |
|  | Validation set TCGA RNA-seq cohort (n = 139) | | |
| Features | Low-risk score (n=69) | High-risk score (n=70) | P-value |
| Age |  |  | 0.075 |
| Mean (range) | 60 (34-85) | 63 (24-89) |  |
| Gender |  |  | 0.881 |
| Female | 25(36.2%) | 25(35.7%) |  |
| Male | 44(63.8%) | 45(64.3%) |  |
| GBM sub-type |  |  | 0.065 |
| Proneural | 12(17.4%) | 12(17.1%) |  |
| Neural | 2(2.9%) | 3(4.3%) |  |
| Classical | 47(68.1%) | 38(54.3%) |  |
| Mesenchymal | 8(11.6%) | 17(24.3%) |  |
| MGMT promoter methylation status |  |  | 0.245 |
| Unmethylated | 33(47.8%) | 34(48.6%) |  |
| Methylated | 24(34.8%) | 19(27.1%) |  |
| NA | 12(17.4%) | 17(24.3%) |  |
| TP53 |  |  | 0.512 |
| Wildtype | 52(75.4%) | 54(77.1%) |  |
| Mutation | 17(24.6%) | 15(21.4%) |  |
| NA | 0(0.0%) | 1(1.4%) |  |
| EGFR |  |  | 0.029 |
| Wildtype | 48(69.6%) | 56(80.0%) |  |
| Mutation | 21(30.4%) | 13(18.6%) |  |
| NA | 0(0.0%) | 1(1.4%) |  |
| Chr 7 gain/Chr 10 loss |  |  | <0.001 |
| Combined | 54(78.3%) | 39(55.7%) |  |
| No combined | 12(17.4%) | 30(42.9%) |  |
| NA | 3(4.3%) | 1(1.4%) |  |

TCGA, The Cancer Genome Atlas; MGMT, methylguanine methyltransferase; TERT, telomerase reverse transcriptase; NA, not applicable; Chr, chromosome.

**Figure S3. Associations between the seven-gene signature and other features in the CGGA and TCGA datasets.**

**
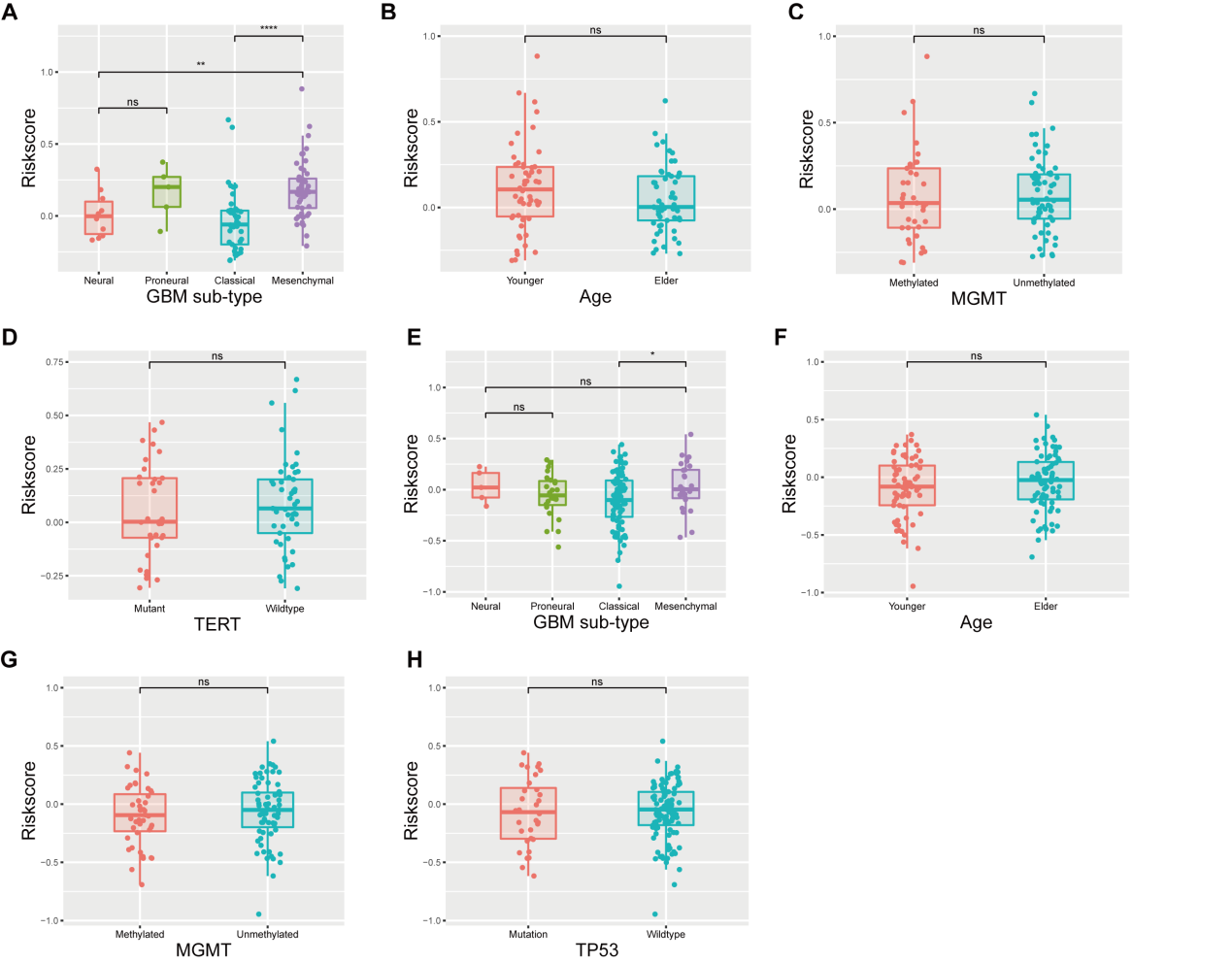
**

**Figure S3. Associations between the seven-gene signature and other features in the CGGA and TCGA datasets. A-D**, Distribution of the seven-gene signature in patients stratified by GBM sub-type **(A)**, age **(B)**, MGMT promoter methylation status**(C)** and TERT promoter status **(D)** in CGGA dataset. **E-H,** Distribution of the seven-gene signature in patients stratified by GBM sub-type **(E)**, age **(F)**, MGMT promoter methylation status**(G)** and TP53 status **(H)** in TCGA dataset. MGMT, methylguanine methyltransferase; TERT, telomerase reverse transcriptase; ***P* < 0.01; *****P* <0 .0001; ns, not significant.

**Figure S4. Functional characteristics related to the seven-gene signature in the TCGA cohort.**

**
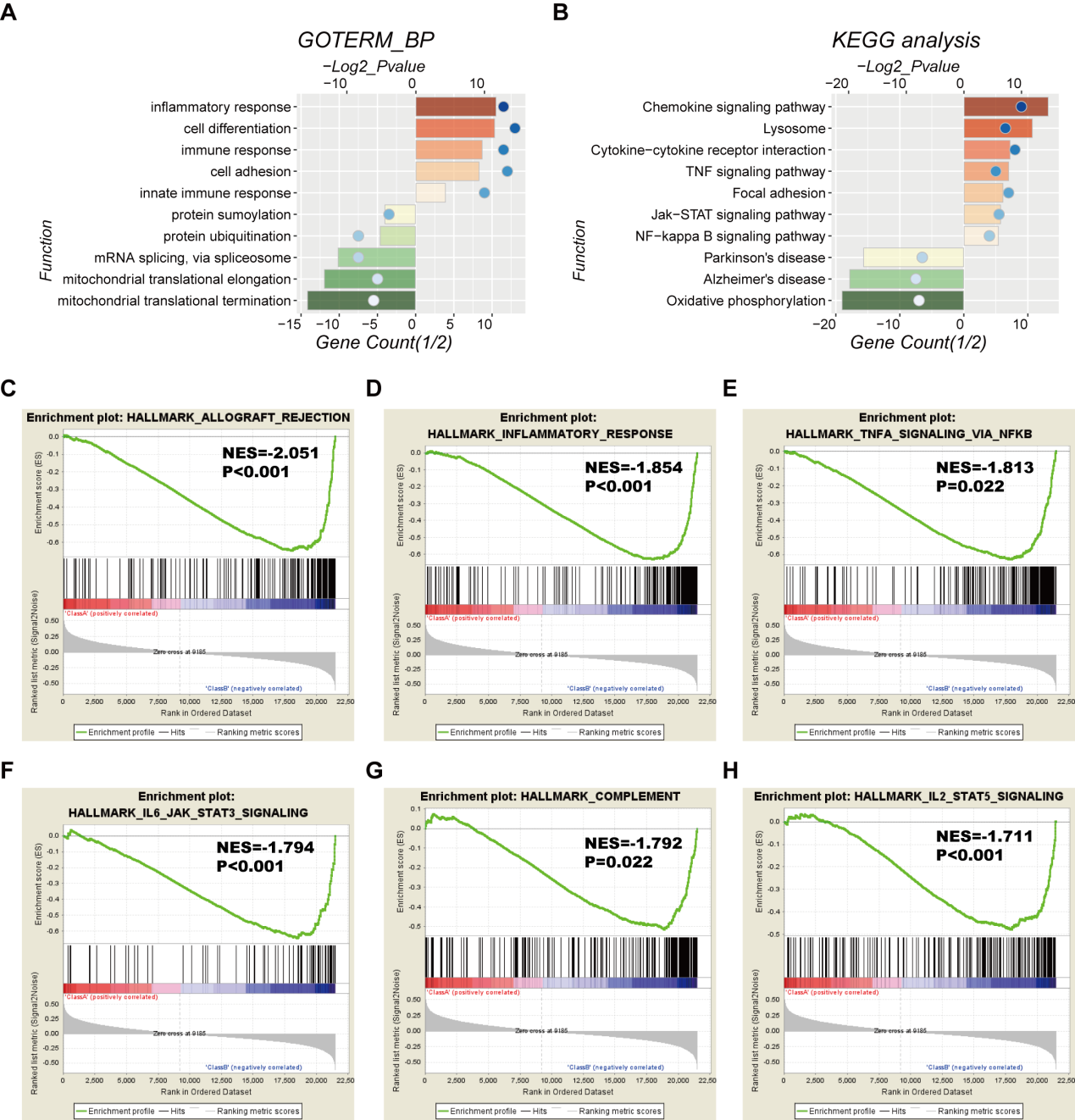
**

**Figure S4. Functional characteristics related to the seven-gene signature in the TCGA cohort. A**,**B**, Functional annotation of genes positively (red bar chart) or negatively (green bar chart) correlated with the risk score using GO terms of biological processes **(A)** and KEGG pathway **(B)**. The orange and green bars represents the *P* value, and the blue dots represent the 1/3 gene count. NES = normalized enrichment score. **C-H**, Gene set enrichment analysis (GSEA) shows that higher risk score was positively associated with inflammatory response, immune response and signaling pathways related to immune response.
